# Demographics, Management Strategies, and Problems in ST-Elevation Myocardial Infarction from the Standpoint of Emergency Medicine Specialists: A Survey-Based Study from Seven Geographical Regions of Turkey

**DOI:** 10.1371/journal.pone.0164819

**Published:** 2016-10-19

**Authors:** Afsin Emre Kayipmaz, Orcun Ciftci, Cemil Kavalci, Emir Karacaglar, Haldun Muderrisoglu

**Affiliations:** 1 Department of Emergency, Baskent University Faculty of Medicine, Ankara, Turkey; 2 Department of Cardiology, Baskent University Faculty of Medicine, Ankara, Turkey; Azienda Ospedaliero Universitaria Careggi, ITALY

## Abstract

**Background:**

This study aimed to explore the ST segment elevation myocardial infarction (STEMI) management practices of emergency medicine specialists working in various healthcare institutions of seven different geographical regions of Turkey, and to examine the characteristics of STEMI presentation and patient admissions in these regions.

**Methods:**

We included 225 emergency medicine specialists working in all geographical regions of Turkey. We e-mailed them a 20-item questionnaire comprising questions related to their STEMI management practices and characteristics of STEMI presentation and patient admissions.

**Results:**

The regions were not significantly different with respect to primary percutaneous coronary intervention (PCI) resources (p = 0.286). Sixty six point two percent (66.2%) of emergency specialists stated that patients presented to emergency within 2 hours of symptom onset. Forty three point six percent (43.6%) of them contacted cardiology department within 10 minutes and 47.1% within 30 minutes. In addition, 68.3% of the participants improved themselves through various educational activities. The Southeastern Anatolian region had the longest time from symptom onset to emergency department admission and the least favorable hospital admission properties, not originating from physicians or 112 emergency healthcare services.

**Conclusion:**

Seventy point seven percent (70.7%) of the emergency specialists working in all geographical regions of Turkey comply with the latest guidelines and current knowledge about STEMI care; they also try to improve themselves, and receive adequate support from 112 emergency healthcare services and cardiologists. While inter-regional gaps between the number of primary PCI capable centers and quality of STEMI care progressively narrow, there are still issues to address, such as delayed patient presentation after symptoms onset and difficulties in patient admission.

## Introduction

ST elevation myocardial infarction (STEMI) is a type of acute coronary syndrome in which coronary plaque rupture, thrombosis, vasospasm, embolization, or dissection leads to complete occlusion of one of the major epicardial coronary arteries, resulting in myocardial injury and necrosis within a period of minutes to hours [1]. It has dynamics and priorities distinct to those of other acute coronary syndromes in that time from coronary occlusion to recanalization in STEMI is significantly correlated to myocardial salvage and viability, ventricular volumes and functions, and long-term development of heart failure and survival [2, 3]. Therefore, studies aimed at optimization of coronary recanalization and revascularization in STEMI from a temporal and technical standpoint has been conducted for a long period of time. Coronary artery recanalization has historically evolved from the chemical degradation of fibrin-bound thrombus by intravenous fibrinolytics [4] to percutaneous techniques (percutaneous coronary intervention, PCI) in which the infarct-related artery is mechanically recanalized [5, 6]. Today, PCI is the gold standard in the treatment of STEMI provided that it is performed in a timely manner and there are sufficient trained staff and equipment [7, 8]. However, fibrinolytics maintain their importance in the management of STEMI, mainly reserved for PCI-incapable centers or whenever time to transfer of patients to PCI-capable centers is delayed or impossible [7, 8].

Emergency departments play a vital role in the proper management of STEMI cases because these patients are mostly transported by emergency transport systems (112, 911, etc) to or seek medical help by themselves at emergency departments of hospitals in Turkey and abroad. Thus, emergency physicians are the first to contact patients with STEMI and perform the important tasks of meeting the critical time window for revascularization, administering the initial treatments such as aspirin, nitrates, and anticoagulants, and referring patients to invasive cardiology or administering fibrinolytics when delay to PCI is anticipated. It is of particular importance how these physicians manage such patients, the medications and revascularization methods they prefer, which difficulties they encounter, and whether they follow the latest state-of-the-art medical practices and updates. It is equally important whether, from the viewpoint of emergency specialists, different regions of our country differ in terms of the management of STEMI cases.

In this questionnaire-based study we aimed to explore the STEMI management practices of emergency medicine specialists working in various healthcare institutions of seven different geographical regions. We also aimed to examine the characteristics of STEMI presentation and patient admissions in these geographical regions.

## Methods

This cross-sectional, observational study was approved by Baskent University Medical and Health Sciences Research Committee and Ethics Committee (Project No: KA15/174).

According to the State Hospitals Statistics Yearbook 2014 [9], the number of emergency specialists working in state hospitals, university hospitals, and private hospitals was 1105. We determined the population size based on this number. We e-mailed a 20-item questionnaire to emergency specialists working in seven geographical regions of Turkey. All respondents completed the questionnaire form according to their daily clinical practice. The participants were allowed to select more than one option for questions 8, 16, and 17. As a result, the total percentage was found above 100% for these questions. The first and the last responses to the survey arrived at 03.06.2015 and 18.06.2015, respectively and kept identities of the participants confidential. We analyzed study data with SPSS for Windows v.17 package software (IBM, Armonk, NY, USA). We compared categoric variables using Exact Pearson Chi-square test. We set the significance level at p<0.05.

## Results

A total of 225 emergency specialists participated in the survey. The median duration of working in this profession was 4 years (range 0–23 years). Thirteen point seven percent (13.7%) (n = 31) of the participants were working in Marmara region, 16.8% (n = 38) in the Aegean region, 14.2% (n = 32) in the Mediterranean region, 16.8% (n = 38) in the Central Anatolian region, 15.1% (n = 34) in the Black Sea region, 12% (n = 27) in the Eastern Anatolian region, and 11.1% (n = 25) in the Southeastern Anatolian region. Survey participation rates were 10.40% in Marmara region, 25.67% in the Aegean region, 26.22% in the Mediterranean region, 16.03% in the Central Anatolian region, 28.09% in the Black Sea region, 28.42% in the Eastern Anatolian region, and 10.40% in the Southeastern Anatolian region. The analysis of the annual number of STEMI patients served in an emergency department showed that 1.8% (n = 4) of the emergency specialists were working at centers with an annual number of patients below 36, 9.8% (n = 22) with 36–70, and 88.4% (n = 199) with above 70. 53.8% (n = 121) of the participants were working at centers with PCI capabilities operating 24 hours a day and 7 days a week, 6.2% (n = 14) were employees of institutions where PCI was only performed during daytime hours of work, and 40% (n = 90) of the participants were working at centers where PCI was not performed. 4% (n = 9) of the emergency specialists preferred to administer fibrinolytics when PCI was not possible at the center, 35.5% (n = 80) preferred to refer patients to PCI-capable centers, and 6.66% (n = 15) referred patients to PCI centers after administering fibrinolytic therapy at the emergency department.

According to the emergency specialist's observations and practices, 66.2% (n = 149) of them stated that patients with chest pain were admitted to the emergency department within between 0 and 120 minutes; 26.2% (n = 59) between 120 and 240 minutes; 6.7% (n = 15) between 240 and 720 minutes; and 0.9% (n = 2) beyond 720 minutes (12 hours). No significant difference was found between centers with vs. without primary PCI capability with respect to emergency department admission times (p = 0.622). According to the participants’ observations and practices, 43.6% (n = 98) consulted with the cardiology department regarding STEMI patients within 10 minutes; 47.1% (n = 106) between 10 and 30 minutes; 8.9% (n = 20) between 31 and 90 minutes; and 0.4% (n = 1) beyond 90 minutes. The medications started by emergency specialists at emergency departments are shown in Table 1.

**Table 1:** Treatments administered by emergency specialists for STEMI patients in emergency department.

Treatment	Number (n)	Percentage (%)
Oxygen (when SpO2 <%90)	214	95.1
Acetylsalicylic acid (ASA)	225	100
Clopidogrel	168	74.7
Ticagrelor	63	28
Prasugrel	28	12.4
Standard heparin (unfractionated—UFH)	97	43.1
Enoxaparin (low-molecular-weight heparin)	133	59.1
Nitroglycerin (sublingual or parenteral)	141	62.7
Proton pump inhibitor (pantoprazole, omeprazole etc)	63	28
H_2_ receptor blocker (ranitidine etc)	45	20
Morphine sulphate	133	59.1
Beta blockers	79	35.1
Angiotensin-converting enzyme inhibitors	30	13.3
Statins	20	8.9
Fibrinolytic therapy	37	16.4
Other	1	0.4

The participants were asked whether they needed to consult regarding STEMI patients with the cardiology department before administering antiplatelet, anticoagulant, or fibrinolytic agents. To this question 20% (n = 45) replied “always”, 15.1% (n = 34) replied “mostly”, 31.6% (n = 71) “rarely”, and 33.3% (n = 75) “never”. The question of whether emergency specialists experienced any problems with patient admission to hospital was answered as “never” by 54.7% (n = 123), “rarely” by 40.9% (n = 92), “mostly” by 2.7% (n = 6), and “always” by 1.8% (n = 4). Sixty-eight percent (n = 153) of emergency specialists stated that the cardiology department always cooperated with them for hospital admissions, 23.6% (n = 53) stated that they mostly cooperated, 4% (n = 9) stated that they rarely cooperated, and 4.4% (n = 10) stated that they never cooperated. Regarding inter-hospital transfers for PCI, 64.4% (n = 145) of the participants said the 112 emergency healthcare services always cooperated with them, 23.6% (n = 53) said they mostly cooperated, 8.4% (n = 19) stated the 112 emergency healthcare services rarely cooperated, and 3.6% (n = 8) stated the 112 emergency healthcare services never cooperated. Among emergency specialists with cardiology staff working at the institution, 83.6% (n = 188) received positive feedback from the cardiology department for the management of STEMI cases. Of the remainder, 12.4% (n = 28) received mixed negative and positive feedback, and only 0.4% (n = 1) emergency specialist always received negative feedback. Three point six percent (3.6%) (n = 8) of the emergency specialists had no cardiology fellows at their institution and therefore they neither refer patients to the cardiology department nor receive any feedback. Thirty one point one percent (31.1%) (n = 70) of the emergency specialists responded “always” to the question of whether quality of STEMI care is affected by the daily number of emergency department admissions, 17.8% (n = 40) responded “mostly”, 25.8% (n = 58) “rarely”, and 25.3% (n = 57) “never”. To the question of whether quality of STEMI care is influenced by the experience and skills of cardiologists and emergency specialists, 55.1% (n = 124) of the participants responded “always”, 20% (n = 45) “mostly”, 13.3% (n = 30) “rarely”, and 11.6% (n = 26) “never, management method is more important”.

Of the emergency specialists, 46.7% opined that personal experience and skills should guide the management of STEMI cases, while others stated that updated guidelines (76%), current studies and expert consensuses (28.4%), and hospital policies and cardiology department’s recommendations (30.7%) should guide practice.

Among the participants, 70.7% stated that STEMI care was carried out according to current guidelines, 46.2% according to personal experience of emergency specialists, 44.2% hospital policy and cardiology department’s recommendations, and 23.6% current studies and expert consensuses.

Forty one point three percent (41.3%) (n = 93) of the emergency specialists were attending scientific meetings, congresses, seminars, or article hours about STEMI care, 5.3% (n = 12) mostly attended these events, 22.7% (n = 51) rarely, and 30.7% (n = 69) never attended such events. A majority (71.6%, n = 161) attended congresses, symposia, or scientific meetings 1–3 times a year, 15.6% (n = 35) attended more than 3 times a year, and 12.9% (n = 29) never attended such events. According to the survey, the daily medical practices of 47.6% (n = 107) of the emergency specialists were influenced by the scientific events attended, 24% (n = 54) stated that their practice principles were rarely altered, and 20% (n = 45) that their practice was mostly influenced by these events. Only 8.4% (n = 18) of the emergency specialists had daily practices unchanged by scientific meetings or events.

**Fig 1:**
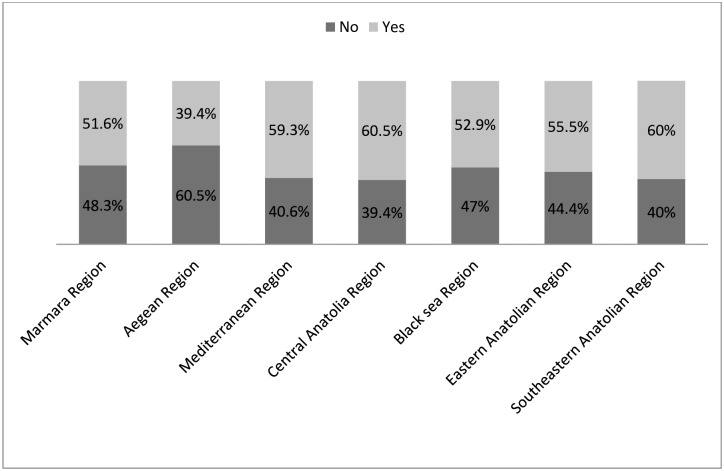
Primary percutaneous coronary intervention rate by geographical region.

**Fig 2:**
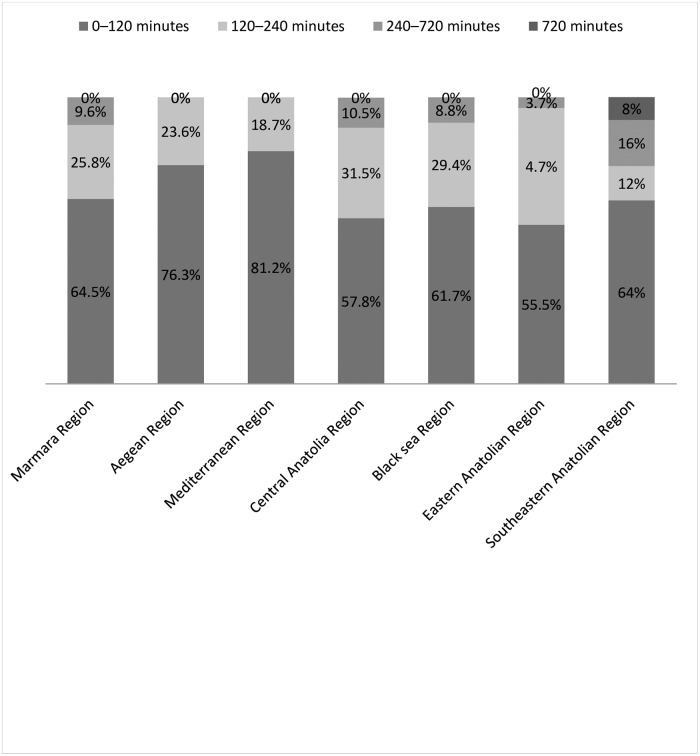
Time from symptom onset to emergency department admission by geographical region.

We have summarized primary coronary intervention availability in STEMI by region in Fig 1. There was no significant difference between the geographical regions with regard to the availability of primary coronary intervention (p = 0.286). However, we detected a significant difference between time from symptom onset to emergency department admission among the different geographical regions (p<0.01) (Fig 2). We also found significant differences between different geographical regions with regard to STEMI patient admission to hospital (p<0.01) (Table 2).

**Table 2:** Frequency of problems related to patient admission by geographical region.

Admission-related problem	Marmara	Aegean	Mediterranean	Central Anatolian	Black Sea	Eastern Anatolian	Southeastern Anatolian	Total	p^a^
**Never**	14 (45.1%)	22 (57.8%)	25 (78.1%)	12 (31.5%)	18 (52.9%)	18 (66.6%)	14 (56%)	123 (54.6%)	<0.01
**Rarely**	16 (51.6%)	15 (39.4%)	7 (21.8%)	24 (63.1%)	14 (41.1%)	7 (25.9%)	9 (36%)	92 (40.8%)	
**Frequently**	1 (3.2%)	0	0	2 (5.2%)	2 (5.8)	0	1 (4%)	6 (2.6%)	
**Always**	0	1 (2.6%)	0	0	0	2 (7.4%)	1 (4%)	4 (1.7%)	
**Total**	31	38	32	38	34	27	25	225	

Values were shown as n (%).

^a^ Exact Pearson Chi-square test.

Twenty-four percent (24%) of the emergency specialists working in the Southeastern Anatolian region stated that their patients mostly presented to emergency departments beyond 4 hours after symptom onset. We noted that the most delayed emergency department admissions were in that region. Eight percent of emergency specialists working in the Southeastern Anatolian region mostly or always had problems related to patient admission to hospitals. This was the highest rate among all regions.

We did not determine any significant inter-regional differences regarding the treatments preferred by emergency specialists in the absence of primary coronary intervention (p = 0.119). Geographical regions were also not significantly different in terms of the cooperation of 112 emergency healthcare services with emergency specialists for inter-hospital STEMI patient transfer (p = 0.064).

## Discussion

This questionnaire-based study evaluated STEMI management by emergency medicine specialists from seven different geographical regions; it also scrutinized geographical differences in STEMI care logistics and patient admission characteristics. To our knowledge, our study is the largest of its kind, reaching 225 of a total of 1105 emergency medicine specialists working in Turkey according to Ministry of Health Statistics [9]. According to the results of the present study, a significant proportion of emergency medicine specialists work at centers with STEMI volumes of more than 70 cases per year, which is in accordance with the data of the Ministry of Health [9]. We believe this is because training in the emergency medicine specialty has relatively recently begun and so emergency departments of university hospitals and training and research hospitals have not been satisfied with the number of available emergency specialists. Accordingly, more than half of the surveyed emergency specialists have been working at PCI-capable centers, of which many have been serving patients with PCI on a 24-hour, 7 days per week basis (24/7). It is encouraging that most PCI centers work on a 24/7 basis since it allows uninterrupted patient management even after hours.

Our study revealed that a significant number of the surveyed emergency specialists refer their patients for immediate PCI either at their own institution or at another, rather than administering fibrinolytics. Some also administer fibrinolytics but only when PCI is not a possibility at the same institution or transfer times exceed the recommended limits; however, they still refer their fibrinolytic-administered patients for routine PCI. Fibrinolytic administration by emergency specialists at emergency departments seems feasible. In contrast, it has also been reported that emergency specialist-administered fibrinolytic treatment may also be not so timely [10]. This subject needs further study.

Our study results showed that a significant majority of STEMI patients present to healthcare facilities within the so-called “golden hour”. This is a satisfactory finding because we know from previous studies that time from symptom onset to presentation and optimal management is inversely proportional to salvaged myocardium and survival, and directly proportional to increased ventricular volumes, cardiac dysfunction, heart failure, and death [11, 12]. Our results indicate that, even when the “golden hour” is exceeded, 99.1% of participants stated patients still fortunately present within the first 12 hours of symptoms, the upper limit of the time interval when primary PCI or fibrinolytic therapy are recommended by guidelines, and the opportunity for revascularization and myocardial salvage remains [13, 14].

Another positive finding of our study is that most cases were discussed with the cardiology department within 30 minutes and almost half within 10 minutes. “Time is muscle” in STEMI [11, 12], and rapid triage, therapeutic decisions, and vigilant monitoring are key to a successful outcome. In our study physicians from only one center reported cardiology referral 90 minutes after admission. The surveyed centers and physicians do not appear to allow time delays in STEMI management.

Regarding medications administered by emergency specialists, all (100%) emergency specialists administer acetylsalicylic acid (ASA) to patients with STEMI as soon as they are admitted with chest pain. It is recommended in all non-aspirin-allergic patients. According to our results, other antiplatelet agents, namely clopidogrel, ticagrelor, and prasugrel were stated to be administered by 74.7%, 28%, and 12.4%, respectively, of the emergency specialists. In our country, prasugrel, and ticagrelor are relatively novel medications as of the time of the survey. Therefore, emergency physicians might prefer using medications they are more familiar with.

Our results showed that emergency specialists do not usually prefer to administer beta blockers, angiotensin-converting enzyme (ACE) inhibitors, or statins in STEMI patients. This may be due to lack of sufficient time for administration before patients are transported or referred to PCI. Swift administration of beta blockers or ACE inhibitors in emergency departments is not necessary [15] and consideration should be given to administer them within the first 24 hours, preferably when a patient is hemodynamically stabilized. It should be strongly emphasized that particularly intravenous beta blockers should not be administered empirically owing to the risk of symptomatic bradycardia, hypotension, shock, pulmonary edema, or even death [16]. It appears logical for emergency specialists not to administer beta blockers and ACE inhibitors in the emergency department. However, statins lack the above mentioned hemodynamic effects and have relatively fewer, if any, side effects. Atorvastatin also possesses pleiotropic effects, namely improvement of endothelial function, inhibition of platelet aggregation, and plaque stabilization in acute coronary syndromes [17, 18], and is recommended at high doses (80 mg) in STEMI [7, 8]. Therefore, a statin administration rate of 8.9% is too low for emergency departments, and statin administration should be given further consideration in this context. Furthermore, the availability of statin should be universalized in the emergency departments.

According to our results, the majority (almost 65%) of emergency specialists do not feel the need to consult the cardiology department regarding administering anticoagulants, antiplatelet agents, or fibrinolytic therapy. This was not surprising since it was noted that they complied with current guidelines and agreed with the statement that emergency specialists should treat patients in compliance with the existing guidelines and state-of-the-art medical codes. We believe that emergency specialists have ever increasing experience and skills in the management of STEMI and access to highly diverse sources of information and communication opportunities to keep them up to date. Studies have shown that such a practice reduces that time and improves outcomes [19, 20].

A great majority of emergency specialists do not experience any problems with patient admissions from emergency departments. We adhere to the Ministry of Health’s hospital bed policies mandating admission of any patient to a vacant bed irrespective of which department that vacant bed belongs to. Additionally, cardiology departments’ eagerness to perform primary PCI, and the special importance hospital administrations attach to the management of STEMI patients are a global indicator of the quality of patient care. Moreover, we did not ask the details of the problems with hospital admission in this survey. We attributed the status of the Southeastern Anatolia region for patient admission to that region’s chronic shortages of equipment, personnel, and logistics.

Our study did not find any difference between geographical regions and the rate of primary PCI or other recanalization techniques. In contrast, patients in the Southeastern Anatolia region seek medical help significantly later than other regions’ residents, such that 24% of emergency specialists working in that region stated that patients presented to them more than 4 hours after symptom onset. This may have several causes, including the harsh conditions, especially in winter, poor transportation, terrorism activities unique to that region, and low socioeconomic status. The exact reason, however, can only be clarified in another study. Similar inter-regional differences may also exist in other countries, albeit to a lesser degree [21–23]. Nevertheless, our task is to abolish or reduce to minimum inter-regional differences in the time from symptom onset to emergency department admission, admission to hospital, and other issues. Our study has some limitations. First, this was a survey-based study and primarily relied on the personal opinions and statements of emergency medicine specialists. We once again stressed the importance of the subjectivity of those times. The study was also primarily qualitative in that we did not use accurate numbers in analyses but ranges and intervals that are simpler to recall by emergency department specialists. Moreover, the participants were not asked about their working institution. We therefore do not know whether more than one physician participated from a single center. To avoid bias, the working institution (private, state, university, teaching) was asked in none of the participants. STEMI was diagnosed based on ECG criteria. However, we did not specifically explored affected heart walls (anterior, inferior etc).

The participants had to choose from among more than one options when asked which treatment option(s) they preferred. The treatment options asked in the survey were not limited to a single patient, but each physician may use separate medications in different patients. Besides, some physicians may administer more than one medication because of lack of appropriate knowledge. We did not include the preferred dosages of the administered medicines in the questionnaire form because the internal medicine specialists were adjusting the dosages according to patient characteristics. Our work did not deal with whether reperfusion preferences of the participants were appropriate.

## Conclusion

Seventy point seven percent (70.7%) of the emergency specialists working in all geographical regions of our country comply with latest guidelines and current knowledge about STEMI care; they also try to improve themselves through participation in scientific meetings, congresses, and seminars. They also receive adequate support from 112 emergency healthcare services and cardiologists. While it seems that inter-regional differences between the rate of primary PCI capable centers and quality of STEMI care progressively narrow, there are still more issues to address, such as delayed patient presentation after symptoms onset and problematic patient admissions.

## Supporting Information

S1 Data The dataset of the study. (XLSX)

S1 Questionnaire The questionnaire form in English and Turkish. (DOCX)
