# Supplementary material for: Demographics, Management Strategies, and Problems in ST-Elevation Myocardial Infarction from the Standpoint of Emergency Medicine Specialists: A Survey-Based Study from Seven Geographical Regions of Turkey
Source: PLoS One. 2016 Oct 19;11(10):e0164819. doi: 10.1371/journal.pone.0164819 (PMC5070734; doi:10.1371/journal.pone.0164819)
Supplement: S1 Questionnaire — (DOCX) [file pone.0164819.s002.docx]

A study of current management approaches of emergency specialists for ST segment elevation myocardial infarction

Emergency departments are the first places where patients with ST segment elevation myocardial infarction are admitted. Taking patients to percutaneous coronary intervention within 90 minutes after first medical contact is considered the preferred management approach where possible.  Emergency specialists assume a critical role in the process of initial management of patients and establishing contact with the cardiology department. We aim to determine working conditions of emergency specialists, the number of STEMI patients they care, and whether emergency specialists practice their profession in accordance with the current STEMI guidelines and the relevant literature.

​ This study was designed to be a collaborative work by *** Emergency and Cardiology Departments. We thank you in advance for your participation in this study.

* Items required to be answered

1) For how many years have you been working as emergency specialist? *

2) In which city do you work? *

3) How many STEMI patients present to your emergency service annually?*

Please mark only one of the choices below

- <36 patients
- 36-70 patients
- >70 patients

4) Is primary coronary angiography available for STEMI patients on a 7/24 basis at your hospital? *

Please mark only one of the choices below

- Yes, it is performed on a 7/24 basis.
- No, it is only performed during working hours.
- No, it is not performed at all.

5) If you answered no to the previous question, what is your treatment plan in such cases?*

(If your answer was "Yes", please mark the last choice

Please mark only one option

- Fibrinolytic therapy
- Referral to a center with coronary intervention capabilities
- Fibrinolytic therapy in emergency department followed by referral for coronary angioplasty
- Primary coronary angiography is available at our hospital

6) According to your observations, how many minutes after chest pain onset do your patients present to emergency service? *

Please mark only one of the choices below

- 0-120 minutes
- 120-240 minutes
- 240-720 minutes
- Beyond 720 minutes (12 hours)

7) According to your observations, how many minutes after emergency department presentation do STEMI patients contact cardiology department?*

Please mark only one of the choices below

- <10 minutes
- 10-30 minutes
- 31-90 minutes
- >90 minutes

8) Which of the following treatments do you administer to STEMI patients at emergency department? *

(You may mark more than one choice)

Please mark all suitable options.

- Oxygen (when SpO2 <%90)
- Acetyl salicylic acid (ASA)
- Clopidogrel
- Ticagrelor
- Prasugrel
- Standard heparin (Unfractionated heparin)
- Enoxaparin (low molecular weight heparin DMAH)
- Nitroglycerin (sublingual or parenteral)
- Proton pump inhibitor (PPI: Pantoprazole, omeprazole etc.)
- H2 receptor blocker (Ranitidine etc.)
- Morphine sulphate
- Beta blocker
- ACE inhibitor
- Statin
- Fibrinolytic therapy
- Other:

9) Do you feel a need for consulting cardiology department before administering antiplatelet, anticoagulant, or fibrinolytic therapy? *

Please mark only one of the choices below

- Yes
- Often
- Seldom
- No

10) Do you experience any difficulty related to patient admissions from emergency department?*

Please mark only one of the choices below

- Never
- Seldom
- Often
- Always

11) Does cardiology department cooperate with you for admissions from Emergency department?*

Please mark only one of the choices below

- Yes
- Often
- Seldom
- No

12) Does 112 coordination center cooperate with you for referrals of STEMI patients? *

Please mark only one of the choices below

- Yes
- Often
- Seldom
- No

13) Does cardiology department provide you with positive or negative feedback regarding your STEMI management?  *

Please mark only one of the choices below

- I get positive feedback. My treatment approaches are supported by cardiology Department
- I get negative feedback. Sometimes I have disagreement with cardiology department regarding my treatment approaches.
- I sometimes get positive and negative feedbacks.
- There is no cardiology expert at our hospital and I am the only caring phyaician for STEMI

14) According to your observations, is the quality of STEMI care affected by emergency department's patient volume? *

Please mark only one of the choices below

- Yes.
- Often.
- Seldom.
- No.

15) According to your observations, is the quality of STEMI care affected by emergency specialist's or cardiologist's professional experience? *

Please mark only one of the choices below

- Yes.
- Often.
- Seldom.
- No. Patient management style is more important than physician experience

16) Which of the following is more important for STEMI care?*

(You may mark more than one choice)

Please mark all suitable choices.

- Personal experience of emergency specialist
- Current guidelines
- Current studies and expert consensuses
- Hospital policy and cardiology department's recommendations

17) At your hospital, according which of the following is the STEMI care done? *

(You may mark more than one choice)

Please mark all suitable choices.

- Personal experience of emergency specialist
- Current guidelines
- Current studies and expert consensuses
- Hospital policy and cardiology department's recommendations

18) Are meetings, presentations, article hours etc. held at your department? *

Please mark only one of the choices below

- Yes.
- Often.
- Seldom.
- No.

19) How many scientific meetings do you attend annually? *

Please mark only one of the choices below

- None
- 1-3
- >3

20) Do scientific meetings affect your daily practice? *

Please mark only one of the choices below

- Yes.
- Seldom.
- Often.
- No/I do not attend scientific meetings.

**Acil Tıp Uzmanlarının ST Segment Yükselmeli Miyokart Enfarktüsüne Güncel Yaklaşımlarının Araştırılması**

ST segment yükselmeli miyokard infarktüsü (STEMI) hastalarının ilk başvuru yeri acil servislerdir. Hastaların ilk medikal teması kurduğu andan itibaren 90 dakika içinde imkân olan merkezlerde perkütan koroner girişime alınması, tedavide öncelikli basamak olarak gösterilmektedir. Acil tıp uzmanları, bu 90 dakikalık sürede tedavinin başlangıcında ve kardiyolojiyle iletişimin sağlanmasında kritik bir görev üstlenmektedir. Çalışmamızda acil tıp uzmanlarının çalışma şartlarını, karşılaştıkları STEMI’lı hasta sayısını, STEMI tedavisinde güncel kılavuzlara ve konuyla ilgili çalışmalara göre hareket edip etmediğini saptamayı amaçlamaktayız.

Çalışmamızı Başkent Üniversitesi Tıp Fakültesi Acil Tıp ve Kardiyoloji Anabilim Dallarının ortak bir projesi olarak gerçekleştirmekteyiz. Anketimize katılımınızdan dolayı teşekkürlerimizi sunarız.

* Yanıtlanması gerekli sorular.

1) Kaç yıldır acil tıp uzmanısınız? * Sayısal Değer:

2) Çalışmakta olduğunuz il hangisidir? * İl:

3) Çalışmakta olduğunuz acil serviste yılda kaç STEMI hastasına bakılmaktadır? *

1. <36 hasta
2. 36-70 hasta
3. >70 hasta

4) Görev yaptığınız hastanede STEMI hastalarına 7 gün/24 saat primer koroner anjiografi yapılmakta mıdır? *

1. Evet, 7 gün/24 saat yapılmaktadır.
2. Hayır, yalnızca hafta içi gündüz, mesai saatleri içinde yapılmaktadır. Mesai saatleri dışında, akşam ve hafta sonu yapılmamaktadır.
3. Hayır, yapılmamaktadır.

5) Bir önceki soruya yanıtınız "Hayır" seçeneklerinden biriyse tedavi planınız ne olmaktadır?*

1. Fibrinolitik tedavi
2. Primer koroner anjiografi yapılan merkeze sevk
3. Acilde fibrinolitik tedavi uygulandıktan sonra primer koroner anjiografi yapılan merkeze sevk
4. Merkezimizde primer koroner anjiografi yapılmaktadır.

6) Gözlemlerinize göre hastalar acil servisinize göğüs ağrısının kaçıncı dakikaları arasında başvurmaktadır? *

1. 0-120 dakika
2. 120-240 dakika
3. 240-720 dakika
4. 720 dakika (12 saat) üzeri

7) Gözlemlerinize göre hastanenizde STEMI hastalarının kardiyoloji bölümüyle ilk teması acil servise gelişinin kaçıncı dakikaları arasında gerçekleşmektedir? *

1. <10 dakika
2. 10-30 dakika
3. 31-90 dakika
4. >90 dakika

8) Acil serviste STEMI hastasına aşağıdaki tedavilerden hangilerini veriyorsunuz? *

(Birden fazla seçeneği işaretleyebilirsiniz)

1. Oksijen (SpO2 <%90 olduğunda)
2. Asetilsalisilik asit (ASA)
3. Klopidogrel
4. Tikagrelor
5. Prasugrel
6. Standart heparin (unfraksiyone-UFH)
7. Enoxaparin (düşük molekül ağırlıklı heparin-DMAH)
8. Nitrogliserin (dil altı ya da parenteral)
9. Proton pompa inhibitörü (PPI: Pantoprazol, omeprazol vb.)
10. H2 reseptör blokörü (Ranitidin vb.)
11. Morfin sülfat
12. Beta blokörler
13. ACE inhibitörleri
14. Statinler
15. Fibrinolitik tedavi
16. Diğer:

9) Acil serviste STEMI hastasına antiplatelet, antikoagülan ve fibronilitik tedavileri başlamadan önce kardiyoloji bölümüyle istişare etme ihtiyacı hissediyor musunuz? *

1. Evet
2. Çoğunlukla
3. Nadiren
4. Hayır

10) Acil servisten STEMI hastasının yatışıyla ilgili problem yaşıyor musunuz? *

1. Hiç yaşamıyorum
2. Nadiren yaşıyorum
3. Çoğunlukla yaşıyorum
4. Her zaman yaşıyorum

11) Acil serviste STEMI hastasının yatışıyla ilgili yaşadığınız problemlerde kardiyoloji bölümüyle işbirliği içinde çalışabiliyor musunuz? *

1. Evet
2. Çoğunlukla
3. Nadiren
4. Hayır

12) Yatışla ilgili yaşadığınız ve hastanın başka bir merkeze sevkini gerektiren problemlerde 112 Komuta Kontrol Merkezi Koordinasyon Birimiyle işbirliği içinde çalışabiliyor musunuz? *

1. Evet
2. Çoğunlukla
3. Nadiren
4. Hayır

13) Acil tıp uzmanı olarak STEMI hastalarını yönetiminizle ilgili kardiyoloji bölümünden olumlu ya da olumsuz geri bildirim alıyor musunuz? *

1. Olumlu bildirim alıyorum. Uyguladığım tedaviler kardiyoloji bölümü tarafından da destekleniyor.
2. Olumsuz bildirim alıyorum. Uyguladığım tedaviler konusunda kardiyoloji bölümüyle çatışmalar yaşayabiliyorum.
3. Kimi zaman olumlu kimi zaman olumsuz geri bildirim alıyorum.
4. Hastanemizde kardiyoloji bölümü veya kardiyoloji uzmanı olmadığı için STEMI hastalarının yönetim ve sevkinden ben sorumluyum.

14) Gözlemlerinize göre STEMI hastasının bakım kalitesi acil servise günlük başvuran hasta sayısından etkilenmekte midir? *

1. Evet, acil servis yoğunluğu STEMI hastasının bakımını olumsuz yönde etkilemektedir.
2. Çoğunlukla etkilenmektedir.
3. Nadiren etkilenmektedir.
4. Hayır, acil servis ne kadar yoğun olsa da STEMI hastasının bakım kalitesi bu yoğunluktan etkilenmemektedir.

15) Gözlemlerinize göre STEMI hastasının bakım kalitesi kardiyoloji veya acil tıp uzmanının kıdeminden etkilenmekte midir? *

1. Evet, etkilenmektedir.
2. Çoğunlukla etkilenmektedir.
3. Nadiren etkilenmektedir.
4. Hayır, etkilenmemektedir. Hekimlerin tecrübesinden ziyade hastayı yönetim tarzı önemlidir.

16) Sizce STEMI hastasının yönetiminde aşağıdakilerden hangisi ön plana çıkmalıdır? *

(Birden fazla seçeneği işaretleyebilirsiniz)

1. Acil tıp uzmanının bireysel tecrübesi
2. Güncel kılavuzlar
3. Güncel çalışmalar ve uzman görüşleri
4. Hastane ilkeleri ve kardiyoloji bölümünün önerileri

17) Çalıştığınız hastanede STEMI hastasının tedavisi aşağıdakilerden hangilerinin doğrultusunda yapılmaktadır? *

(Birden fazla seçeneği işaretleyebilirsiniz)

1. Acil tıp uzmanının bireysel tecrübesi
2. Güncel kılavuzlar
3. Güncel çalışmalar ve uzman görüşleri
4. Hastane ilkeleri ve kardiyoloji bölümünün önerileri

18) Çalıştığınız bölüm içinde STEMI ile ilgili toplantı, sunum, makale saati gibi etkinlikler yapılıyor mu? *

1. Evet
2. Çoğunlukla
3. Nadiren
4. Hayır

19) Yılda kaç kez kongre, sempozyum gibi bilimsel toplantılara katılıyorsunuz? *

1. Hiç katılmıyorum
2. 1-3 kez
3. >3 kez

20) Katılmış olduğunuz bilimsel toplantılar günlük pratiğinizi etkiliyor mu? *

1. Evet, etkiliyor.
2. Nadiren etkiliyor.
3. Çoğunlukla etkiliyor.
4. Hayır, etkilemiyor/veya bilimsel toplantılara katılmıyorum.
